# Chinese vocational college students pursuing a baccalaureate: academic motivation and career adaptability

**DOI:** 10.3389/fpsyg.2026.1849938

**Published:** 2026-06-16

**Authors:** Zhong-xing Wang, Yue Liu

**Affiliations:** 1 Yantai Vocational College, Yantai, China; 2 Shandong Port Polytechnic College, Yantai, China

**Keywords:** academic motivation, baccalaureate attainment, career adaptability, career decision-making, higher vocational education

## Abstract

**Introduction:**

The present study aimed to explore how academic motivation (AM) and career adaptability (CA) affect baccalaureate attainment among Chinese higher vocational students.

**Methods:**

An explanatory sequential mixed-methods approach was applied to the research design. A sample of 1,563 participants was surveyed with a questionnaire, and in-depth interviews were conducted with 33 participants.

**Results:**

The results indicated that more than half of the participants (59.2%) decided to apply for a baccalaureate, whereas the rest decided to work (23.5%) or remained undecided (17.3%). The main findings are as follows: female students, Grade Point Average (GPA) at an average level, and students from low-income families intended to apply for a baccalaureate. Quantitative analyses revealed that extrinsic motivation was the primary predictor of baccalaureate intention, while the concern dimension of career adaptability showed a significant but negative association with this choice. Extrinsic-oriented utility motivation exceeds intrinsic motivation as the main source of academic transfer. Remedial support is essential for students to pass the transfer examination.

**Discussion:**

These results have important policy implications for Chinese universities receiving transfers from higher vocational colleges regarding student retention and academic success.

## Introduction

Chinese post-secondary education has evolved into a hierarchical system, with higher vocational education serving as the lowest tier for students with low academic performance (Liu, 2013; Liu and Wang, 2015). Unlike universities, Chinese vocational colleges generally offer a 3-year diploma and do not confer bachelor’s degrees (Hansen and Woronov, 2013). Higher vocational students are “left-over” (Zha, 2012) due to their poor performance in the National College Entrance Examination (CEE). Like students in the US community colleges, Chinese vocational graduates can choose to transfer to a university to earn a baccalaureate. Whereas these graduates must pass a closed-book provincial examination called College to University Transfer Examination (CUTE), and the acceptance rate is low. In recent years, the number of vocational students choosing to transfer has increased dramatically (Mycos Institute, 2021). A statistical report on China’s educational achievements in 2021 showed that a total of 4,460,700 high school graduates were enrolled in higher educational institutions. Among them, 717,700 students transferred from higher vocational colleges to universities, representing a 7.66% increase over previous years (MOE, 2022).

Extant studies have demonstrated that vocational education can help reduce youth and structural unemployment (Rageth and Renold, 2020; Watson, 1994). However, it remains less attractive to students with academic education in China. Students’ perceptions of their future play an essential role in career development. The disparity of demographic factors also affects the academic and career trajectories of youngsters. Some studies have shown that students with low academic performance were not well prepared as their counterparts (Flores, 2003; Thernstrom and Thernstrom, 1996). Ethnic and racial variables were regarded as key factors to influencing intentions to pursue higher education (e.g., Slobodin et al., 2021; Frings et al., 2020). Some studies indicate that vocational students often have low academic and socioeconomic backgrounds (Ainsworth and Roscigno, 2005; Guo and Wang, 2020). Although vocational education could be advantageous for early employment opportunities, its advantages diminished when observed across many countries (Hanushek et al., 2017). Baccalaureate attainment could enhance employability by acquiring advanced knowledge and skills and by avoiding the devaluation of the degree due to credential inflation (Waters, 2009). It reflects that the realization of vocational education should shift from an employability approach to a career approach that meets individuals’ development needs.

The present study aims to examine the role of academic motivation (AM) and career adaptability (CA) in shaping Chinese higher vocational students’ intentions to pursue a baccalaureate degree. Specifically, this study investigates: the motivational profiles that drive students’ transfer decisions; the dimensions of career adaptability associated with these decisions; and the demographic and academic factors that predict students’ career intentions.

The following three research questions are formulated:

1. What motivates Chinese higher vocational college students to pursue a baccalaureate?
2. How do Chinese higher vocational college students make their career decisions?
3. What predicting factors affect motivation and career adaptability among Chinese higher vocational college students?

## Literature review

As outlined in the introduction, this study is grounded in self-determination theory (SDT) and career construction theory (CCT) and is situated within the broader decision-making frameworks of planned behavior and push-pull dynamics.

Planned behavior theory (Ajzen, 1991) posits that attitude, subjective norm, and perceived behavioral control influence intention, whereas intention, with or without perceived behavioral control, may lead to actual behavior. Under the guidance of the planned behavior approach, there are extant studies to disclose students’ intentions. Ingram et al. (2000) found that attitude is the only predictor of the intention to apply to graduate school. Tan and Laswad (2006) found that parents had a deep influence on students’ intentions to major in accounting.

The push–pull model (Ravenstein, 1885) was initially used to study population movement and was later extended to research on how behavior is “organized” or “planned.” Wen and Hu (2019) demonstrated that the push–pull model has been widely used to investigate potential factors influencing student mobility. Li and Bray (2007) found that family background, individual academic ability, and students’ genuine aspirations to study abroad significantly influenced their decisions and motivations to study in Hong Kong and Macau. Bodycott and Lai (2012) found that personal interest in a new educational and cultural context affects students’ choices. Moreover, the experiences of family members and peers significantly affect students’ motivation. The push–pull model reveals that multiple external factors—social, economic, political, environmental, and even geographical—affect students’ study choices. It is an effective tool for depicting the process of career decision-making, providing theoretical ground for how predisposition or intent to study is formulated.

### Academic motivation

Self-determination theory (SDT) was regarded as an underpinning theory of academic motivation, providing the fundamental theoretical framework for the study of academic or learning motivation (Deci and Ryan, 2002). It emphasizes the role of intrinsic and extrinsic motivation in driving behavior. SDT posits that individuals are most motivated when their psychological needs for autonomy, competence, and relatedness are satisfied (Deci and Ryan, 2002). Within this framework, amotivation refers to the absence of intentional regulation, wherein individuals lack the motivation to act or feel disconnected from the outcomes of their actions (Deci and Ryan, 2002). While SDT has been widely applied in educational settings, there is a need to further extend it to develop a more comprehensive theory of academic motivation. Schunk et al. (2014, p. 36) identified motivation as “an energized internal state that results in goal-directed behaviors.” Students highly anticipated that higher education would accompany with prosperous career, more opportunities, and financial security (CCCSE, 2016; Mayhew et al., 2016; O’Banion, 2013). Extant studies have shown that students’ motivations play an essential role in academic outcomes across different educational levels (e.g., Frings et al., 2020; Gegenfurtner, 2011; Vansteenkiste et al., 2010).

Although motivation can be regarded as an essential personal trait in pivotal life decision-making, it also varies in the context of higher education, with its opportunities and challenges (Briggs et al., 2012; Christie et al., 2008). Academic motivation has been defined in a domain-general and domain-specific manner. Vansteenkiste et al. (2006) categorized learning motivation as autonomous motivation and controlled motivation. *Autonomous motivation* comprises intrinsic motivation (e.g., sincere interest regardless of outcome) and identification (e.g., own regulation because of personal relevance); *Controlled motivation* consists of introjected (e.g., internal pressure to pursue self-worth or avoid guilt and shame) and external regulation (e.g., rewards and punishments) (Deci and Ryan, 2002; Vansteenkiste et al., 2006). These motivational orientations are distinctly related to students’ academic achievement (Datu et al., 2018; Guay et al., 2010; Ratelle et al., 2007). In particular, autonomous motivation is linked to adaptive academic outcomes, whereas amotivation is associated with indicators of maladaptive academic functioning (Datu, et al., 2018; Guay et al., 2010; Ratelle et al., 2007). Controlled motivation is connected with positive academic outcomes (Datu et al., 2018).

According to these theories and findings, academic motivation is rarely explained by a single perspective. Individuals differ not only in the quantity of their motivation but also in its direction and type (Chevrier and Lannegrand, 2022). One common issue in educational systems is low academic motivation among students, which leads to significant educational, cultural, and economic issues (Turner and Reed, 2022). Some studies have shown that academic motivation is related to vocational education. Despite the imbalance between intrinsic and extrinsic motivation, vocational students believed school was important for their future success (Lee-Genzel and Bekir, 2024). Vocational students’ academic motivation declined in their first year of school but remained stable in subsequent years (van der Veen and Peetsma, 2020).

### Career adaptability

Career theories (Super, 1957) have developed concepts such as vocational identity, career planning, career development, and career stages, which are used to predict adjustment and behavior in work or learning environments. To help adolescents answer the question “what should I do for a better future” or to find the right “life trajectory,” some theoretical explorations and practical attempts are emerging. Savickas et al. (2009) proposed that the life-designing paradigm is an effective career intervention that endorses five of the preceding presuppositions (i.e., context, process, non-linear dynamics, narrative realities, and modeling) about people. Their work lives: contextual possibilities, dynamic processes, non-linear progression, multiple realities, and personal patterns. Accordingly, the aim is to improve adaptability.

Career adaptability first emerged as a construct within life-span, life-space theory, and was later applied to the life-designing approach (Levin and Lipshits-Braziler, 2021; Super, 1990; Savickas et al., 2009). Career construction (Savickas, 2002, 2013) provides a developmental perspective that refines four dimensions: (a) concern about the future, (b) control over their lives, (c) curiosity about occupational careers, and (d) confidence to construct a future and deal with career barriers. *Concern* is a positive action for the future that may yield contradictory results. If the concern is based on personal experience, practice, and active activities, it will bring hope and the future opportunities. If the concern stems from insufficient attention or excessive preoccupation with the results, it will be detrimental to individuals’ ability to make a rational decision. *Control* is an active action of self-adjustment. Effective control can enhance self-regulation and responsibility, building a mindset of autonomy and self-reliance; insufficient control makes individuals feel wavering when dealing with the future uncertainty. *Curiosity* reflects an inquisitive attitude, which encourages individuals to pursue a flexible career and make educational choices. Risk-taking and inquiring behaviors are regarded as the essential strategies for coping with the future undecided issues. *Confidence* attributes contribute to problem-solving ability and self-efficacy, which encourage individuals to compare different coping strategies to achieve optimal results. To conclude, career adaptability can be regarded as the ability, through effective coping strategies, to make optimal decisions in the face of uncertainty. Levin and Lipshits-Braziler (2021) asserted that career adaptability was verified to depict the career-decision-making process and reported that career adaptability “concern,” decision-making adaptability “procrastination,” and “speed of making the final decision” were significant predictors of decisional difficulty.

Career adaptability plays a crucial role in vocational education by helping students navigate career-related challenges. Recent research highlights its interaction with academic motivation, particularly in competency-based learning environments. For instance, students with higher career adaptability exhibit stronger intrinsic motivation, as they view education as directly relevant to their future careers (Rudolph et al., 2021). This adaptability fosters resilience, enabling learners to persist despite academic difficulties (Guan et al., 2023). Conversely, students with low adaptability may rely more on extrinsic motivation (e.g., grades or parental pressure), which can reduce long-term engagement (Negru-Subtirica et al., 2021). Vocational education settings amplify this relationship, as career-specific training enhances perceived competence—a key motivator in self-determination theory (Ryan and Deci, 2020). When students see clear career pathways, their adaptability strengthens, reinforcing mastery goals over performance-based ones (Hirschi et al., 2022). Interventions like career counseling and experiential learning further boost both adaptability and motivation (Johnston et al., 2023). Thus, career adaptability and academic motivation interact dynamically in vocational education, with adaptability fostering self-regulated learning while motivation sustains career exploration—a cycle critical for workforce readiness. However, as this study will show, the empirical contribution of career adaptability to understanding transfer decisions remains limited in certain contexts, warranting further theoretical integration with constructs such as the future-oriented thinking and sustainable career beliefs (La Rosa, and Zammitti, 2025).

## Methodology

### Participants

The quantitative data were drawn from a larger study in which 1,563 students were enrolled voluntarily from a Chinese vocational college in Shandong province and were informed about the purpose of the study, data confidentiality, and the right to withdraw at any time. As Table 1 shows, participants are males, 41.7%; females, 58.3%; first-grade students, 34.4%; second-grade students, 28.0%; and third-grade students, 37.6%. Participants were from the following majors: Mechatronics Engineering (15.2%), English (18.7%), Informational Technology (19.5%), Accounting (20.5%), and Early Childhood Education (26%). Their family incomes were distributed as follows: 47.1% for below 40,000 yuan, 16.3% for 40,000–60,000 yuan, 20% for 60,000–100,000 yuan, 9.1% for 100,000–140,000 yuan, and 7.5% for over 140,000 yuan. For the qualitative part, snow sampling is repeated until the required number (*N* = 33) is reached for a qualitative in-depth interview.

**Table 1:** Distributions of career decision-making.

Count (% within)		To work	Apply for a degree	Not decided	Total
Gender	Male	163 (44.3%)	348 (37.6%)	141 (52.2%)	652 (41.7%)
	Female	205 (55.7%)	577 (62.4%)	129 (47.8%)	911 (58.3%)
Grade	First	71 (19.3%)	362 (39.1%)	104 (38.5%)	537 (34.4%)
	second	78 (21.2%)	263 (28.4%)	98 (36.3%)	439 (28.1%)
	Third	219 (59.5%)	300 (32.4%)	68 (25.2%)	587 (37.6%)
Major	Mechatronics engineering	50 (13.6%)	128 (13.8%)	59 (21.9%)	237 (15.2%)
	English	58 (15.8%)	205 (22.2%)	30 (11.1%)	293 (18.7%)
	Information technology	79 (21.5%)	152 (16.4%)	74 (27.4%)	305 (19.5%)
	Accounting	64 (17.4%)	217 (23.5%)	40 (14.8%)	321 (20.5%)
	Early childhood education	117 (31.8%)	223 (24.1%)	67 (24.8%)	407 (26.0%)
Family income	Below 40,000 Yuan	191 (51.9%)	407 (44.0%)	138 (51.1%)	736 (47.1%)
	40,000–60,000 Yuan	53 (14.4%)	152 (16.4%)	50 (18.5%)	255 (16.3%)
	60,000–100,000 Yuan	71 (19.3%)	202 (21.8%)	40 (14.8%)	313 (20.0%)
	100,000–140,000 Yuan	29 (7.9%)	89 (9.6%)	24 (8.9%)	142 (9.1%)
	Above 140,000 Yuan	24 (6.5%)	75 (8.1%)	18 (6.7%)	117 (7.5%)
GPA	Poor	19 (5.2%)	17 (1.8%)	5 (1.9%)	41 (2.6%)
	Below average	35 (9.5%)	47 (5.1%)	30 (11.1%)	112 (7.2%)
	Average	203 (55.2%)	478 (51.7%)	158 (58.5%)	839 (53.7%)
	Above average	67 (18.2%)	272 (29.4%)	48 (17.8%)	387 (24.8%)
	Excellent	44 (12.0%)	111 (12.0%)	29 (10.7%)	184 (11.8%)
Total		368 (23.5%)	925 (59.2%)	270 (17.3%)	*n* (100%)

### Tools

*Chinese version of the modified career adapt-abilities scale (CAAS):* some demographic variables (e.g., gender, family income, major, and academic performance) and intentions of the future plan (i.e., “to work,” “to pursue a baccalaureate degree” or “have not decided yet”) emerged in CAAS (Savickas and Porfeli, 2012), which is composed of 24 items in four sub-dimensions: concern, control, curiosity, and confidence. Items were marked using a 5-point Likert scale (1 = Very untrue of me; 5 = Very true of me). The CAAS was translated from English into Chinese by two bilingual researchers using a forward-backward translation procedure. Discrepancies between the original and back-translated versions were resolved through discussion until consensus was reached. The Cronbach’s alpha coefficients of concern, control, curiosity, and confidence were 0.89, 0.90, 0.88, and 0.86, respectively. Confirmatory Factor Analysis (CFA) indices showed a good fit to the data: Comparative Fit Index (CFI) = 0.902, Tucker-Lewis’s index (TLI) = 0.908, Root Mean Square Error of Approximation (RMSEA) = 0.046, and Standardized Root Mean Square Residual (SRMR) = 0.053. These values met the standard criteria (CFI ≥ 0.90, TLI ≥ 0.90; RMSEA ≤ 0.08) (Hu and Bentler, 1999), indicating adequate model fit (see Table 2).

**Table 2:** Indices of CFA for CAAS.

	X^2^/df	RMSEA	CFI	TLI	SRMR
Indices value	3.894	0.046	0.902	0.908	0.053
Threshold	<5.000	<0.08	>0.90	>0.90	<0.08

*Chinese version of academic motivation scale – college (AMS-C)*: AMC-C (Vallerand et al., 1992) was adopted, consisting of 28 items. There are three sub-dimensions: intrinsic motivation, extrinsic, and amotivation. Items were marked using a 5-point Likert scale (1 = Very untrue of me; 5 = Very true of me). All these questions and items are translated into Chinese. The Cronbach’s alpha coefficients of intrinsic motivation, extrinsic motivation, and amotivation were 0.87, 0.86, and 0.81, respectively. CFA indices values showed a good data fit: CFI = 0.922, TLI = 0.918, RMSEA = 0.036, SRMR = 0.061. These values met the standard criteria (CFI ≥ 0.90, TLI ≥ 0.90; RMSEA ≤ 0.08) (Hu and Bentler, 1999), indicating adequate model fit (see Table 3).

**Table 3:** Indices of CFA for AMS-C.

	X^2^/df	RMSEA	CFI	TLI	SRMR
Indices value	4.036	0.053	0.922	0.918	0.061
Threshold	<5.000	<0.08	>0.90	>0.90	<0.08

*Interview protocol*: the semi-structured interview protocol presented in this study will outline a comprehensive set of questions to confirm or further interpret the quantitative findings. The interview protocol was designed participants to be centered on the main constructs in AMS-C: how to know, accomplish, and experience stimulation among intrinsic motivation (questions like “is there any individual disposition motivates you to apply for a baccalaureate”), how to identified, introjected and external regulated among extrinsic motivation (questions like “is there any external factors motivate you to apply for a baccalaureate”), and what are the source of amotivation (questions like “what makes you be nonsense of future plane”). The protocol questions also be assigned to elicit the constructs of CAAS: concern (questions like “have you ever concerned about future”), control (questions like “have you ever adopt some strategies to accomplish your plane”), curiosity (questions like “are you ever feel curious about the future which may change your life dramatically”), and confidence (questions like “are you confident about your decision-making of future”).

### Data analysis

A mixed-methods design was conducted as a fully mixed sequential equal-status type (Leech and Onwuegbuzie, 2009), which included two phases: First, a quantitative survey with a questionnaire was administered, and Statistical Package for the Social Sciences (SPSS) version 23.0 was used for data analysis. In this process, Cronbach’s alpha reliability coefficients and descriptive statistics (i.e., mean and standard deviation) for all variables were calculated, and correlational and multinomial regression analyses were conducted to examine the association between career adaptability and academic motivations. All items were screened for missing values, outliers, and normality. Missing data were addressed using multiple imputation, and outliers were identified using Mahalanobis distance (Enders, 2010). Online data collection occurred over 1 month, using a secure research platform for study information, electronic consent, and completion of self-report questionnaires. A multinomial logistic regression was selected because the dependent variable—career decision-making intention—has three unordered categories (to work, to apply for a baccalaureate, and undecided). In this model, the following variables were treated as categorical predictors: gender (male as the reference), GPA level (excellent as the reference), and family income (above 140,000 yuan as the reference). All career adaptability and academic motivation sub-scale scores were treated as continuous variables, as they were measured on 5-point Likert scales. The decision to treat motivation and adaptability scores as continuous is consistent with the standard practice in the literature for Likert-scale composite scores (e.g., Savickas and Porfeli, 2012; Vallerand et al., 1992). The reference category for the outcome variable was “undecided,” as this group represented students who had not yet formed a clear career intention and thus provided a meaningful baseline for comparison. Second, data was collected by thorough semi-structured interviews. Potential participants were targeted based on demographic distribution and quantitative data, with clear criteria for purposive sampling and efforts to ensure demographic representativeness. The initial purposive selection was guided by two core criteria: consistency with the demographically meaningful variables (gender, GPA, family income, major, and year of study) identified in the quantitative phase, and possession of typical research-related experiences to ensure data richness. To ensure representativeness, the 10 initial participants were selected to cover diverse genders, academic years (freshman to senior), major categories (humanities, social sciences, and natural sciences), three GPA tiers (low, medium, and high), and different family income levels, reflecting the target population’s diversity. Another 23 students were recruited via snowball sampling through introductions from first-round participants. Sampling stopped at data saturation, operationally defined as the absence of new core themes, viewpoints, or research-related information emerging from three consecutive interviews. Three qualitatively experienced researchers reviewed and independently analyzed the data, meeting regularly to discuss emergent issues and themes and to reach consensus (Vaismoradi et al., 2016). Participants (*N* = 33) were asked to answer questions based on quantitative results. The questions included “Have you ever designed your future career?” “What motivated you to choose apply for a baccalaureate or go to work directly after graduation?” etc. The transcriptions of the audio-taped interviews were analyzed using a four-step thematic analysis process. This part aimed to be reflexive during the analysis and not confer ideas on the data (Patton, 2002). In the first step, we strived to get a holistic sense of the data without sorting or coding. In the second step, descriptive phrases that pertained to participants’ academic motivations and career adaptability were extracted. For example, “I prefer to apply for a baccalaureate after exploring different trajectories” (Participant 5) and “The reason why I intend to apply for a baccalaureate is that my parents wanted” (Participant 11). In the third step, initial codes for the data were generated by segmenting and labeling the extracted phrases. The codes were categorized into the dimensions of motivation and adaptability. For example, Participant 11’s description was coded in the category of extrinsic motivation; Participant 5’s description was coded in the category of curiosity. In the fourth step, codes were synthesized as themes through paraphrasing. For example, participants 3, 7, and 11 (P3, P7, and P11) asserted that parents, relatives, or friends motivated them to apply for a baccalaureate. After peer debriefing and discussion, “significant others” emerged as a theme depicting the academic motivation of Chinese high vocational students.

## Findings

Over half of the participants (59.2%) chose the option of applying for a bachelor’s degree (see Table 1) after graduation, and the proportions of participants who chose to work (23.5%) or who were undecided (17.3%) were close. Among the respondents who chose to work were comparatively high proportions of females (55.7%) and third-year students (59.5%), as well as students who had an average GPA (55.2%), a low family income (51.9%), and majored in informational technology (21.5%). Among the respondents who chose to apply for a bachelor’s degree were comparatively high proportions of females (62.4%), as well as students who had average GPA (51.7%), had low family income (44.0%), were first-year (39.1%), or majored in early childhood education (23.5%). Undecided respondents contained comparatively high proportions of males (52.2%) and first-year students (38.5%), as well as students who had a low family income (51.1%), majored in informational technology (27.4%) and had an average GPA (58.5%). Based on Table 3, average GPA and low family income are the main groups with higher proportion among various future plan options.

### Quantitative results

*The correlations between career motivation and career adaptability*: the means, standard deviations, and Pearson correlations for academic motivation sub-dimensions and career sub-dimensions are presented in Table 4. It shows that internal motivation is positively correlated with concern (*r* = 0.55, *p* < 0.01), control (*r* = 0.64, *p* < 0.01), curiosity (*r* = 0.67, *p* < 0.01), confidence (*r* = 0. 68, *p* < 0.01), and external motivation (*r* = 0.89, *p* < 0.01). External motivation is positively correlated with concern (*r* = 0.59, *p* < 0.01), control (*r* = 0.62, *p* < 0.01), curiosity (*r* = 0.65, *p* < 0.01), and confidence (*r* = 0.67, *p* < 0.01). Amotivation is positively correlated with control (*r* = 0.11, *p* < 0.05) and curiosity (*r* = 0.09, *p* < 0.05); negatively correlated with internal motivation (*r* = −0.26, *p* < 0.01) and external motivation (*r* = −0.18, *p* < 0.01). Amotivation is not correlated with concern and confidence. The high correlation between intrinsic and extrinsic motivation (r = 0.89) raises concerns about multicollinearity, which is addressed in the regression analysis below.

**Table 4:** Descriptive statistics and bivariate correlational coefficients among the variables.

Variable	Mean	SD	1	2	3	4	5	6	7
1. Concern	3.75	0.68	1	–	–	–	–	–	–
2. Control	3.84	0.67	0.75^**^	1	–	–	–	–	–
3. Curiosity	3.91	0.70	0.70^**^	0.84^**^	1	–	–	–	–
4. Confidence	3.94	0.69	0.69^**^	0.85^**^	0.651^**^	1	–	–	–
5. I. M.	3.72	0.69	0.55^**^	0.64^**^	0.67^**^	0.68^**^	1	–	-
6. E. M.	3.83	0.67	0.59^**^	0.62^**^	0.65^**^	0.67^**^	0.89^**^	1	-
7. Amotivation	2.88	0.94	0.037	0.11^*^	0.099^*^	0.090	0.-25^**^	−0.18^**^	1

*p < 0.05; **p < 0.01. IM, intrinsic motivation; EM, extrinsic motivation.

*Multinomial regression on students’ future intention*: given the high correlation between intrinsic and extrinsic motivation (r = 0.89), variance inflation factors (VIF) were examined and found to be within acceptable limits (all VIF < 5.0). The overall model was statistically significant (χ^2^ = 245.67, df = 28, *p* < 0.001), with Nagelkerke’s pseudo-R^2^ = 0.19. As Table 5 shows, compared with participants who had not decided what to do in the future, those who chose to work were at a low level of amotivation (OR = 0.770), a negatively high level of internal motivation (OR = −1.716), and a low family income (OR = 1.394). In comparison to the participants who had not decided what to do in the future, those who intended to apply for a baccalaureate were in a low level of concern (OR = 0.836), a negatively high level of internal motivation (OR = −1.783), a high level of external motivation (OR = 1.237), a high level of GPA average (OR = 1.315), and a high level in low family income (OR = 1.361).

**Table 5:** Multinomial regression on students’ career intentions.

Predicators	To work		To apply for a baccalaureate	
	OR	SE	OR	SE
Concern	0.712	0.350	0.836	0.312*
Control	0.062	0.495	0.607	0.416
Curiosity	−0.320	0.578	−0.508	0.466
Confidence	0.325	0.577	0.148	0.493
IM	−1.716	0.585*	−1.783	0.482*
EM	0.611	0.574	1.237	0.473*
Amotivation	0.770	0.198*	−0.047	0.140
GPA level				
Poor	2.195	1.141	0.533	1.436
Below average	−0.104	0.691	−0.613	0.619
Average	−0.134	0.552	1.315	0.444*
Above average	0.118	0.625	0.566	0.493
Gender (Female as reference)	−0.317	0.324	−0.623	0.272
Family income (above 140,000 as a reference)				
Below 40,000	1.394	0.661*	1.361	0.524*
40,000–60,000	1.090	0.708	1.368	0.554
60,000–100,000	0.853	0.701	0.979	0.543
100,000–140,000	1.380	0.886	1.794	0.707

*p < 0.05; “Have not decided” as the reference of the future intention.

### Qualitative results

Semi-structured interviews were conducted, and the guidelines were prepared using theory-based, rule-guided method (Gläser and Laudel, 2010). Initially, open coding was conducted by segmenting and labeling raw interview transcripts with descriptive tags (e.g., “degree skepticism,” “parental influence,” and “financial anxiety”). This inductive phase allowed themes to emerge organically without predetermined categories. Through constant comparative analysis, codes were refined and grouped into broader conceptual clusters (e.g., “credential struggler” and “significant others dependent”). Axial coding was then employed to examine relationships between categories—for instance, how external pressures (e.g., job market demands) conflicted with internal ambivalence. To ensure analytical rigor, peer debriefing was used to challenge interpretations, while member checking verified whether participants recognized their experiences in the preliminary findings. After thematic analysis, seven themes emerged: credential struggler, significant others, reality rebel, examination revenger, money subservience, injustice escaper, and social expectation conformist (see Table 6).

**Table 6:** Descriptions of main themes emerging from qualitative study.

No.	Themes	Motivation	Adaptability	Examples
1	Credential struggler	Amotivation: With no choice	Concern: Aware of value	“I have no sense of future. Although credential is valueless, studying is the only way I am familiar with. It is the easiest choice for me” (Participant 4)
		Extrinsic: Identified to prepare for a career	Concern: Aware of value	“There is an electronic products manufactory nearby my home. It is said that associate degree holders could be recruited last year but baccalaureate. Holder required this year. You know, this job can only earn 4,000 RMB per month, even if I cannot get it if I cannot get a baccalaureate” (Participant 1)
2	Significant others dependent (1) family members	Extrinsic: Driven by others	Control: Take family responsibility	“My mother always complains that she always bears the discrimination that my credential is inferior to that of her relatives and friends. She told me that if I can apply for a baccalaureate, she will be proud of me” (Participant 11)
		Intrinsic: To know some new things	Confidence: Learn skills	“My parents own an international business company. I will inherit it in the future. I need to learn new skills and at least get a baccalaureate. to be capable to run the business” (Participant 2)
	(2) antecedents	Extrinsic: Driven by others	Control: Make up your mind	“When I entered college, one senior encouraged me to apply for baccalaureate and share her successful experience with me. Then I decided to follow her” (Participant 3)
	(3) off-campus institutions	Extrinsic: Driven by others	Control: Make up your mind	“Some off-campus institutions enter our college to promote their course I am poor of self-control but still want to apply for baccalaureate. it is said that more than 80% of their course takers can pass the examination, so I decided to pay for these courses” (Participant 10)
3	Reality rebel	Amotivation: Capricious	Curiosity: Explore differences	“I am not satisfied with the status quo, not satisfy with anything. I want to change all, such as my major, my school, but I am sure this change is valuable or correct, just change. In order to change, I apply for baccalaureate” (Participant 14)
4	Examination revenger	Extrinsic: introjected	Concern: Realizing dreams	“I really regretted that when I have not studied hard in high school. I do not want to be a failure again, so the transfer examination is the last opportunity for me” (Participant 8)
5	Money subservience: money works	Extrinsic: External regulations	Concern: Aware of values	“Get a higher degree is the proximity to a decent job. I am always thinking of how to avoid a labor work although it can earn more money. Sometimes, I do prefer the work in office with little money because it may provide me more opportunities to be promoted” (Participant 9)
	(2) money not works	Extrinsic: External regulations	Concern: Aware of values	“Money is the main consideration to support your dream. I know some students with high academic performance giving up to apply for a baccalaureate. Just because of lack of money. Her parents want her to work directly to share the family economic burden” (Participant 13)
6	Injustice escaper	Extrinsic: External regulation	Concern: Avoiding deficiency	“Female students are vulnerable in labor market, because we cannot handle some labor jobs. Only office works suit us. In order to get this kind of job, we must get a baccalaureate” (Participant 6)
7	Social expectation conformist	Extrinsic: External norms	Concern: Avoiding discrimination	“My high school classmates all went to four-year universities. At every reunion, I feel embarrassed when they talk about their ‘real’ college experiences while I’m stuck with an associate degree. Even if I hate exams, I’d rather suffer through two more years than face their pity” (Participant 33)

*Credential struggler*: pursuing a baccalaureate degree is a mechanical behavior because they are unsure whether a degree can change their fate. However, they are sure that in the job market, not having a degree will put them in trouble. Their decision reflects a self-contradiction, in that the absence of internal motivation yields to external reality.

- “I am not sure that to apply a BA is worthy. But I have no choice because most of companies will not accept me if I just hold an associate degree. How can I get through of it?” (Participant 7).
- “It takes me long time to decide whether to apply for a baccalaureate. On one way, I do not like to study. On the other way, I need to get a secure job. Reality finally defeats my internality” (Participant 4).

*Significant others dependent*: this group saw significant others (i.e., parents, friends, and teachers) as the key source to decide what to do in the future.

- “It is not easy for my mother to raise me. She told me to continue studying, and I think I should listen to her, which is the best way to show my filial piety” (Participant 5).
- “I really appreciate the senior’s advice. One of them has offered the university information and the previous examination papers to me, which she collected when she prepared for enrollment last year. Furthermore, she instructed me on the application strategies to avoid fierce competition to achieve success” (Participant 11).

*Reality rebel*: the decision-making is unconscious, impulsive, and even blind because they do not know the career trajectories. Their personal traits play an essential role in their choices.

- “I know that even got a baccalaureate or master degree, it is still hard to find a satisfying job. I just want to be in a good mood, which I assume that another university may confer it to me” (Participant 2).
- “I know that school time is most enjoyable period in life. Extending schooling time is my initiative to apply for a baccalaureate. I hate the “complicated” relationships in workplace, which makes me unhappy” (Participant 14).

*Examination revenger*: these participants regarded the examination as the most essential tool to change their fate. They usually thought their scores on the College Entrance Examination did not reflect their actual level of academic ability.

- “The competition of CEE is really fierce, which is nicknamed a single-log bridge through which the only road towards success. I would like to regard associate-to-baccalaureate degree examination or postgraduate examination as the last straw of success” (Participant 8).
- “My father had died just 2 months before CEE. I was totally in sorrow and hardly concentrate on my study, so I failed. At present, I need to rebound to study hard to pursue higher education for a bright future” (Participant 10).

*Money subservience*: students take financial status as an important reference for their future choices. For money and for the sake of money are the roots of study or work.

- “Earning more money is my priority when I graduated because I came from a poor family. If applying for a baccalaureate, I must persuade my parents to pay for it. It is really a hard process. Only get permission from my parents, I can totally embed in preparation” (Participant 9).
- “I am poor of self-control but still want to apply for a baccalaureate. Paying for the cost enhances your impetus. The cost of extra courses is really expensive, about 12,000 RMB. So, I must study hard” (Participant 4).

*Injustice escaper*: various forms of discrimination in the Chinese labor market and testing system prompt students to adapt by adopting avoidance the situation strategies. Students who perceive locus of control as more internal may be more determined to be resilient when faced with external demands and obstacles.

- “The testing system is biased. The subjects of exams are English, mathematics, and ideology and politics. These courses only taught in the first year of college. However, our examination holds on the third year when I almost forgot all. In that case, we need extra courses” (Participant 5)
- “Female graduates were vulnerable in labor market, which pushes us to apply for higher degree to get a job without depending on physical labor. We are avoiding some jobs instead of choosing some jobs” (Participant 6)

*Social expectation conformist*: this group feels compelled to pursue a baccalaureate due to societal pressures, even if they lack personal interest. They perceive higher education as a societal norm rather than a personal aspiration.

- “In my hometown, people always ask, “Which university are you attending?” If I only have an associate degree, they’ll look down on me. Even if I do not like studying, I have to meet their expectations—otherwise, I’ll lose face” (Participant 21).
- “All my cousins have bachelor’s degrees. My grandparents keep comparing me to them. I do not want to be the ‘black sheep’ of the family, so I must get this degree, no matter how useless I think it is” (Participant 25).

### Mixed-methods integration

The integration of quantitative and qualitative data is essential for mixed methods research (Creswell and Plano Clark, 2011). Consistent with the mixed-methods case study design, we merged the quantitative results with the qualitative findings to understand the case better. Specifically, we compared the emergent themes with survey responses to develop findings as follows: (1) Quantitatively high proportion of students pursuing a baccalaureate who are qualitatively varied in academic motivations; (2) quantitatively more females pursuing a baccalaureate who have suffered from gender discrimination; (3) quantitatively GPA average students pursuing a baccalaureate who qualitatively get remedial support from off-campus institutions; and (4) quantitatively low family income students pursuing a baccalaureate who qualitatively regarded the degree as a utility to get a fruitful job (see Table 7).

**Table 7:** Joint display table of quantitative and qualitative findings.

Integrated dimension	Quantitative data	Qualitative findings
Academic motivation	59.2% of students pursue a baccalaureate degree	Varied motivations and career adaptability
Gender disparity	62% female enrollment in baccalaureate programs	Extrinsic motivation: External regulation Adaptability of concern: Avoiding deficiency
Academic performance	58.6% applicants’ GPAs are not above average	Extrinsic motivation: driven by others Adaptability of control: make up your mind
Family income	44% of applicants’ family income is below 40,000 RMB annually	Extrinsic motivation: External regulations Adaptability concern: Aware of values

The mixed-methods integration reveals two complementary patterns that deepen the understanding of the quantitative findings. First, although 59.2% of students intended to pursue a baccalaureate degree, the qualitative data indicate that this intention is driven by diverse motivational profiles rather than a single pathway. For instance, students with average GPAs were more likely to pursue a baccalaureate, but the qualitative findings explain this pattern through the “significant others dependent” and “examination revenger” themes: these students often rely on off-campus remedial institutions and external encouragement to compensate for academic under-preparedness. Second, the quantitative finding that low family income predicts baccalaureate intention is illuminated by the “money subservience” theme, which reveals a dual financial logic: students from low-income families view the degree as a tool for economic mobility, yet simultaneously experience financial anxiety that nearly prevents them from pursuing it. This tension between aspiration and constraint is not visible in the regression coefficients alone. Third, the gender disparity in baccalaureate pursuit (62% female) is contextualized by the “injustice escaper” theme, which shows that female students perceive the degree as a protective mechanism against labor market discrimination rather than as a positive career development strategy. Finally, the career adaptability dimension of concern emerged as the sole significant predictor among the four CAAS sub-scales, and the qualitative data suggest that this reflects a reactive, avoidance-oriented concern (e.g., “avoiding deficiency”) rather than proactive career planning. This distinction may explain the unexpected negative coefficient for concern in the regression and points to the need for a more nuanced conceptualization of career adaptability in this population.

The quantitative correlation between academic motivation and career adaptability is depicted qualitatively as the interactions: (1) extrinsic motivation interacted with concern and control; (2) intrinsic motivation interacted with curiosity and confidence; and (3) amotivation interacted with concern and confidence.

## Discussion

The present study indicated that more than half (59.2%) of the students preferred to apply for a baccalaureate after high vocational education, which is in accordance with previous findings on postgraduate students in the Chinese educational context (e.g., Liu and Morgan, 2016). Although the quantitative data did not reflect a significant disparity in academic motivation, the qualitative data showed that the motivation of higher vocational students to apply for a baccalaureate was mostly extrinsically driven. Highly academically performing students have not yet been regarded as the main cohort to pursue a baccalaureate, which is contrary to previous studies (e.g., Guay et al., 2010; Kusurkar et al., 2013; Ratelle et al., 2007). Academically average students were highly motivated to apply for a baccalaureate in the first year of higher vocational education but less motivated in subsequent years. However, this finding should be interpreted with caution, as the cross-sectional design does not permit direct inferences about motivational change over time; longitudinal data would be required to confirm this pattern. That means a transfer “shock”—academic under-preparedness (Xu et al., 2018; Zamani, 2001)—affects the motivations of students who may be culturally, socially, and psychologically prepared for transfer, yet not academically, cognitively, and meta-cognitively prepared. Therefore, so-called “baccalaureate aspirants” could be depicted as academically average students with less learning interest and strategy but relying on extrinsic motivation, which they regarded as the last straw to help them achieve success. The seven qualitative themes identified in this study reveal that baccalaureate aspirants are not a monolithic group; rather, they represent distinct motivational profiles ranging from externally driven compliance (e.g., “social expectation conformist”) to reactive escape (e.g., “injustice escaper”) to internal exploration (e.g., “reality rebel”). These profiles carry different implications for post-transfer academic success.

Female students are highly motivated to pursue a baccalaureate degree, exhibit more positive academic behaviors, and have more holistic expectations for their education (Wawrzynski and Sedlacek, 2003). Some interviewees answered that female workers were vulnerable and had fewer opportunities because they are disadvantaged in physical labor. Extending study time is one of the key strategies to combat gender discrimination (Yue and Qiu, 2022). Family income did not emerge as a factor affecting students’ academic motivation, which is inconsistent with previous studies (Craig, 2019).

Aside from money perceived as the key consideration for their future careers, students also admitted that various social and family perceived supports (e.g., mortgage and parental income) push them to move forward. Despite low academic motivation, the students’ choice of choosing to apply for a baccalaureate is more a form of procrastination in career decision-making. Some interviewees responded that applying to a baccalaureate program was a secondary choice because the few optimal options were available to them. This is an avoidance strategy to escape from the fierce competition in the labor market. In addition, career adaptability, as a set of strategies or processes, reflects the behavioral dispositions of Chinese vocational college students. The processes of students’ decision-making were mainly in “concern” and “control” (e.g., comparing with different choices; being aware of values), while “curiosity” and “confidence” (e.g., attempting and exploring the options) processes were regarded as essential components of successful career decision-making (Betz et al., 1996; Taylor and Betz, 1983). This interpretation aligns with recent work by Russo et al. (2023), who found that young adults’ sustainable career beliefs and the future-oriented thinking are influenced not only by immediate external pressures but also by broader cognitive and motivational resources. Similarly, La Rosa and Zammitti (2025) demonstrated that emerging adults’ perceptions of sustainable careers are shaped by both future time perspective and environmental concern, suggesting that career adaptability in vocational populations should be conceptualized as a multidimensional construct interacting with personal future orientation, a dimension that the present study did not fully capture but that warrants attention in the future research.

The significant others theme has emerged as one that has not yet been included in the main framework of academic motivational measurement. In China, influenced by Confucian culture (Liu and Lu, 2011), youngsters rely more on significant others (e.g., parents, relatives, friends, and teachers) in their career and academic choices, exhibiting traits such as “filial piety,” which is also the core embodiment of collectivism. In addition, gender discrimination makes the decision disproportionate and hesitant (Yue and Qiu, 2022). For theoretical framework construction, AMS-C (Vallerand et al., 1992) extrinsic motivation should include items reflecting items of the specified environment and social norms to disclose behavioral dispositions across various contexts.

Academic motivation is utility-driven by external factors rather than by a desire to acquire knowledge and self-accomplishment among Chinese higher vocational students. In contrast, amotivated students choose to pursue a baccalaureate when no better options are available, as it offers an expedient way to escape a state of having no other choices. “What is valuable?” is the most frequently asked question, which alludes to the students on the path of making a career decision. In their minds, a degree is a facilitator for earning more money, a leverage for a happy marriage, and recognition for entering office work. Bers and Galowich (2002) studied the influence of parents on college choice for students attending a community college in an affluent suburb. Parents indicated that financial factors were more influential than college reputation. Remedial or extra instruction provides convenience for those who are not excellent in GPA to apply for a higher degree. Previous research indicated that remedial interventions appear to promote persistence and degree completion (Pascarella and Terenzini, 2005), whereas other studies suggested that enrolling in remedial courses has a negative effect on completing a degree (Adelman, 1999; Bailey and Alfonso, 2005). As some respondents described, “Nearly 90 percent of us enrolled in extra courses because it is extremely effective, although it costs above 10,000 RMB. But it is really worthwhile. Furthermore, students can hardly pass the examination without paying for these courses” (Participant 11).

The constructs of motivation in AMS-C are positive psychological behaviors oriented toward depicting academic motivation as achieving, realizing, accomplishing, and identifying. By contrast, the qualitative data disclosed some negative forms of academic motivation to fight against, to counter, to rebel for identities and discrimination. The gap between quantitative and qualitative results will prompt the integration of both positive and negative perspectives when designing further measurement tools. That means negative psychology and passive behaviors might serve as the source of positive motivation.

## Implications of the present study

### Implications for theoretical development

**Figure 1:**
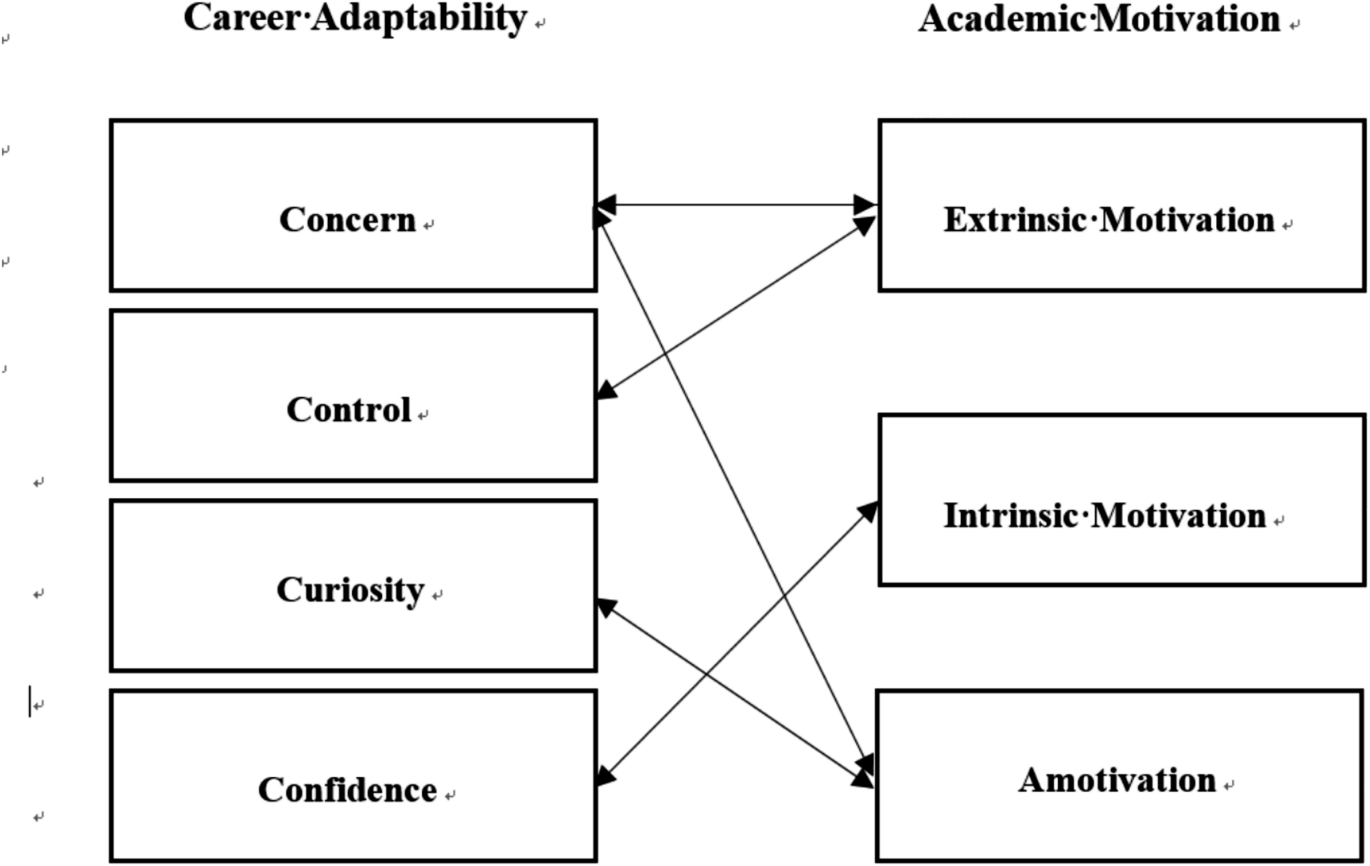
The integrated framework of CA and AM.

The present study explored the mechanism underlying baccalaureate attainment among Chinese vocational college students by integrating theories of motivation and adaptability. The bidirectional arrow (see Figure 1) between the two constructs represents the reciprocal relationship identified in both the quantitative and qualitative findings: career adaptability dimensions—particularly concern—were associated with students’ motivational orientations. At the same time, extrinsic motivation emerged as the primary driver shaping how students exercised adaptive strategies in their career planning. This reciprocal relationship suggested that students’ proactive engagement with the future career prospects enhances their motivation to pursue higher education. In contrast, external incentives (e.g., job opportunities and societal expectations) play a crucial role in strengthening their adaptability skills in the Chinese context. Furthermore, this framework requires further verification to ensure its validity across diversified contexts.

Previous studies (Hoxby and Turner, 2023; Bukodi and Goldthorpe, 2024) have shown that high socioeconomic status (SES) students are more likely to obtain higher degrees. However, the present study found that low SES students were also willing to pursue higher degrees. The findings showed that external motivations, including economic, cultural, and peer influences, also lead to students from disadvantaged groups to have enthusiasm for a baccalaureate. This reveals that academic guidance and institutional support are very crucial. It is necessary to help students understand career choices before they enter university and make rational decisions. Meanwhile, colleges should offer more support, such as academic tutoring, to help students pursue a baccalaureate degree.

### Implications for practice

Findings from the present study highlight several important implications for educational policy and practice. The fact that GPA is not a predictor of baccalaureate attainment and persistence elicited reflections on the defining role of the academic experience at Chinese higher vocational colleges in shaping transfers’ academic outcomes. From the perspectives of policymakers and college or university leaders, improving student learning and academic performance may be the most effective way to promote desirable student outcomes. Remedial interventions should be organized not only off-campus but also within the curriculum for prepared students. The lack of academic interest among baccalaureate aspirants who are just pursuing utility may hinder their attainment by the end of a 4-year university program. Academic climate is the key solution to assisting students in making rational and long-term decisions. Career consulting and intervention should nurture their adaptability so they can follow the best trajectory for the future development.

The gender gap in baccalaureate attainment among higher vocational transfers implies that 4-year institutions hosting these transfers should not label this student group as a homogenous sub-population. University administrators should build an inclusive culture to ensure that when students enter midway. Policies should promote students’ learning interests, so they can quickly adapt to the new curriculum, and practices should provide more perspectives for students’ future selection. Therefore, it is helpful for administrators and faculty at 4-year institutions to consider demographic differences among higher vocational transfers in this relearning process and form policies and strategies accordingly.

Structured career guidance and targeted policy interventions can significantly enhance vocational students’ rational decision-making regarding pursuing a bachelor’s degree. Germany’s Career Orientation Program integrates aptitude tests and industry visits to align further education with labor market needs. At the same time, Australia’s My Future platform provides data-driven employment trend insights—models China could adapt by mandating vocational guidance courses in year 1 and developing artificial intelligence (AI)-powered decision tools. The disparities in attainment based on career decisions generate policy and practical issues for a “humanity”-oriented education approach whose main function is to leverage diverse career and academic trajectories. By the end of December 2022, the Chinese Ministry of Education had issued “a guideline to advance reform and high-quality development of vocational education.” It reaffirmed the positioning of vocational education, which is to serve the all-round development of people, establish and improve the gradient vocational education and training system with multi-form cohesion, multi-channel growth and sustainable development, promote the coordinated development and mutual integration of vocational and general education, so that students with different endowments and needs can make multiple choices and become diversified talents, which will reverse the social disdain for vocational education. Furthermore, more vocational colleges will gain the right to grant baccalaureate degrees to baccalaureate aspirants.

### Implications for the future research

Although academic motivation and career adaptability are closely interrelated, baccalaureate attainment and college persistence among Chinese higher vocational transfers are ultimately distinctive processes shaped by different combinations of influences. While the proposed conceptual model predicts baccalaureate attainment reasonably well, an expanded and improved model needs to be developed to study transfer student persistence in the future research. The present study utilizes mixed methods to provide integrated empirical research to illuminate the future study of the broader theoretical frameworks and approaches, such as career theory and behavior theory, with the extension of the education discipline.

Some factors, such as SES, have not emerged either in quantitative or qualitative data to be predictive for students’ decision-making. Recent studies indicated that social capital, parental expectations, and policy support were factors shaping educational aspirations. Students embedded in peer networks where classmates plan to pursue further education were more likely to follow suit (Li and Zhang, 2023). Parental pressure to attain higher credentials remained strong, even for average-achieving students (Chen and Huang, 2024). The government further incentivized this transition by offering employment-linked benefits, such as preferential hiring for bachelor’s graduates in public-sector jobs (Zhou, 2022). As the higher vocational transfer student population continues to diversify and new initiatives to fill the social-economic gap are introduced, more research needs to be conducted to identify effective models on the basis of a sociopsychological approach, as well as optimal measures in assessing the effectiveness of academic choices. Career theory is the key foundation for further studies aimed at enhancing education outcomes. Research on the process and quality of decision-making should be integrated into a comprehensive model to improve the wellbeing of youngsters. This requires extant longitudinal studies to emerge, learning, and working experience. A sample college limits the generalizability of the findings. Therefore, the number of sample colleges should be expanded in the future studies.

## Limitations

One limitation is the strong link to the local context, which makes the findings directly relevant to that context but less generalizable to others. Moreover, the cross-sectional design does not permit causal inferences about the relationships between academic motivation, career adaptability, and career intentions, nor does it allow for the examination of changes in motivation over time. Longitudinal studies are needed in the future research.

## Conclusion

The present study examines the trends and characteristics of Chinese higher vocational students’ decision-making regarding the pursuit of a baccalaureate. The main findings indicate that female, average-GPA, and low-income students intend to apply for a baccalaureate. Two emerging themes depict the motivation and its correlation with career adaptability. Among them, extrinsic-oriented utility motivation exceeds intrinsic motivation as the main source of academic transfer. Remediation is essential for students to pass the transfer examination. These results have important policy implications for universities receiving transfers from higher vocational colleges regarding student retention and success. The findings of this study should also be of interest to policymakers at the state level in expanding the cooperation and collaboration among all institutions of higher education and related stakeholders, and more importantly, formulating well-informed policies to assist students in accomplishing baccalaureate learning to get a best future.

## Data Availability

The datasets presented in this study can be found in online repositories. The names of the repository/repositories and accession number(s) can be found in the article/supplementary material.
